# Recurrent Nevus Phenomenon Developing within a Keloid

**DOI:** 10.3390/dermatopathology10030028

**Published:** 2023-06-30

**Authors:** Cody J. Rasner, Yan Zhou, Alessio Giubellino

**Affiliations:** Department of Laboratory Medicine and Pathology, University of Minnesota, Minneapolis, MN 55455, USA; zhou1237@umn.edu

**Keywords:** recurrent nevus, recurrent nevus phenomenon, keloid, atypical melanocytic proliferation

## Abstract

The recurrent nevus phenomenon represents the persistence of a nevus within a scar from a prior biopsy site, with the acquisition of clinical and histologic features frequently overlapping with those of melanoma, posing relevant diagnostic challenges. Similar features are recognized in nevi that have undergone recent or chronic trauma and in sclerosing nevi. Any type of nevus may be subject to this phenomenon. Keloids are exuberant scars with an exaggerated accumulation of dense dermal collagen. Here we report a case of a 42-year-old woman with the incidental finding of an atypical melanocytic proliferation developing within a keloidal scar. The patient presented with a progressively enlarging auricular lesion three years after a piercing procedure. Upon histological examination, attentive scrutiny of the margin revealed an atypical compound melanocytic proliferation, predominantly single-celled at the junction but occasionally nested, with cytologic atypia and architectural disorder. This atypical proliferation was found emerging above a keloid. We interpreted the lesion as an atypical melanocytic lesion with features resembling the recurrent nevus phenomenon. This case raises awareness in recognizing these melanocytic lesions as benign, thereby avoiding overdiagnosis and unnecessary treatment.

## 1. Introduction

The recurrent nevus phenomenon is described as the reappearance or persistence of a nevus within the scar from a prior excision due to the benign proliferation of remaining melanocytes [1,2,3]. Any type of nevi (e.g., congenital, dysplastic, blue-type) may occur in this phenomenon following excision [4,5,6,7]. Recurrent melanocytic lesions appear to affect middle-aged females more commonly, are most frequently found on the back followed by the face and extremities, and usually develop within 6 months of the original biopsy [5]. This latency is usually in contrast with the recurrence of a melanoma at the same site, which may develop after several months or even years following the re-excision [8,9]. Similar features of atypical melanocytic proliferations may occur in another context; for example, following various injuries with traumatic scars [5,10,11].

The exact mechanism leading to the development of recurrent nevi remains unclear. One potential hypothesis is connected to the activity of the various growth factors released by fibroblasts and other cell types in the local tissue after the excision of a nevus. These growth factors are involved in tissue repair and subsequent scar development. However, once released in the tissue microenvironment, they may exert additional functions as, for example, incidentally inducing the proliferation of junctional and dermal melanocytes, possibly leading to an aberrant melanocytic proliferation. However, the precise interplay between fibroblasts, melanocytes, and other cells is not well understood, and this represents an active field of research [2,3,11].

Keloids are essentially hypertrophic scar tissue that are characterized by an aberrant fibroproliferative response. On histology, they are characterized by dense, hyalinized bands of collagen [12]. Trauma or disruption of the basement membrane is considered one of the earliest events in keloid formation; it is hypothesized that this disruption triggers a proliferation signaling cascade, resulting in the extension of collagenous tissue beyond the original margins of damaged tissue. Keloids are associated with increased pigmentation, yet there is a severe lack of data supporting melanocyte involvement in keloid development [12]. Reports of both benign and malignant melanocytic lesions developing within a keloid are scant in the literature [12,13,14]. 

Here, we present a case of a melanocytic lesion developing within a keloid scar of a female patient who initially sought medical attention for cosmetic concerns related to an expanding growth on her ear. However, upon careful histological analysis of the excised tissue, an unexpected finding of an atypical melanocytic proliferation within a keloid scar was revealed. Further evaluation by dermatopathologists confirmed that the melanocytic proliferation was benign and exhibited histological characteristics consistent with that of the recurrent nevus. 

By the traditional definition, the recurrent nevus requires the presence of a pre-existing nevus. However, melanocytic lesions histologically identical to the recurrent nevus may occur in the absence of any pre-existing lesion. Alternative nomenclature previously described in the literature include “pseudo-melanoma” or “persistent nevus”, although the use of the term “pseudomelanoma” has been debated more heavily in the literature as it is potentially misleading as the term is also used in describing hemosiderotic histiocytoma [2]. For the purposes of this case report, we use the term recurrent nevus phenomenon as this is the most widely used term; however, it is unclear whether a melanocytic lesion existed at this site prior to the development of the keloid. 

The identification of the recurrent nevus phenomenon within a keloid scar in this case underscores the importance of dermatopathologists and dermatologists considering this phenomenon. Cases such as this may pose significant diagnostic challenges in determining the malignant potential. In cases where patients present with keloid scars and exhibit concerning pigmented changes, clinicians should consider the possibility of a benign melanocytic proliferation within the scar tissue, even in the apparent absence of a primary nevus. 

## 2. Case Description

A 42-year-old otherwise healthy female presented with a progressively enlarging 1 cm by 1 cm skin lesion that was slightly hyperpigmented, wrinkled, and raised, developing over the helical rim of her left ear. No erythema, warmth, purulence, ulceration, or other indications of infection were present. The patient ultimately presented for cosmetic indications and otherwise had no systemic symptoms including no fever, rash, weight loss, or other concerning signs of systemic illness or malignancy. The patient underwent a piercing procedure approximately 3 years earlier at the exact site; no other trauma was reported. With the provided history of the piercing trauma and with the clinical evaluation of a dermatologist, the lesion was presumed to be a keloid scar. 

**Figure 1:**
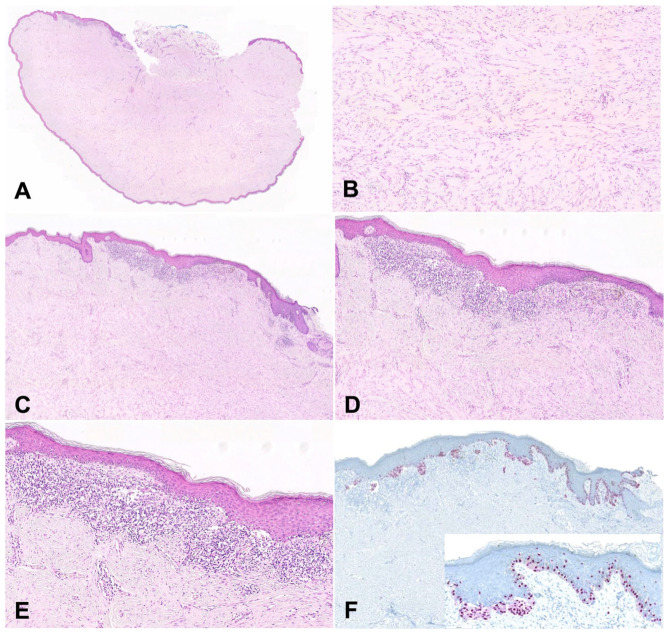
(**A**) Histology of the entire keloid with the location of the atypical melanocytic proliferation (10×). (**B**) Representative histology of the underlying keloid scar (400×). (**C**) Low-power image of the atypical compound melanocytic proliferation (100×). (**D**,**E**) High-power image showing a single-celled area with mild cytologic atypia and cells in pagetoid array (400×, 1000×). (**F**) Immunohistochemical staining for SOX10 highlights the lesion (100×; inset: 400×).

Given the progressive enlargement of the lesion, surgical excision was performed. The tissue was sent for histological evaluation, revealing a dense nodular aggregate of fibrosis with keloidal collagen (Figure 1), consistent with a keloid, confirming the clinical impression. On one biopsy margin of the keloidal tissue, there was an atypical compound melanocytic proliferation characterized predominantly by single-celled but occasionally nested melanocytic proliferation, with cytologic atypia and architectural disorder. 

**Figure 2:**
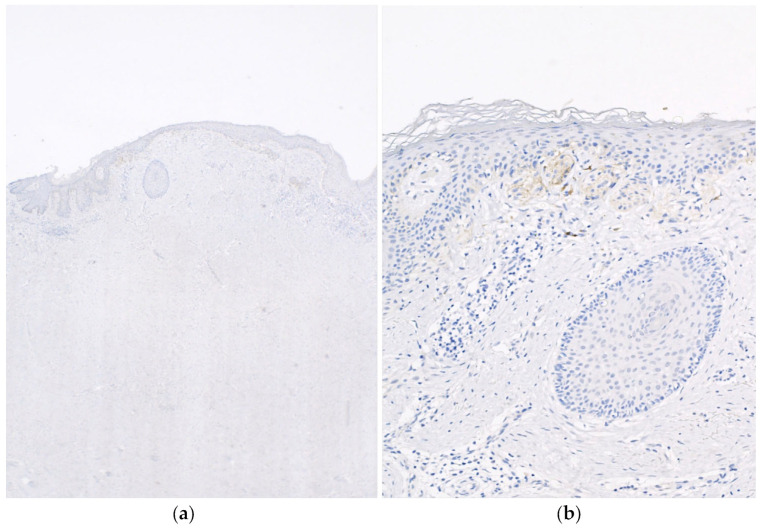
(**a**) Low-power image of the histology of the keloid with the area of the overlying atypical melanocytic proliferation after the PRAME stain (40×). (**b**) PRAME staining was negative (no nuclear staining) throughout the lesion (400×).

No high-grade atypia or mitoses were present (Figure 1). Given the presence of the lesion above the scar tissue, the lesion was suspected to represent a melanocytic proliferation with features resembling the recurrent nevus phenomenon. The tissue was subsequently stained for SOX10, which revealed single-celled melanocytes exhibiting a pagetoid spread within the epidermis as well as nested melanocytes with mild atypia. Subsequently, a PRAME (preferentially expressed antigen in melanoma) stain was performed and was negative (no staining, Figure 2), supporting the interpretation of a benign lesion. 

Following the surgical excision and diagnosis, a close clinical follow-up was recommended for the patient. To date, no recurrence or further interventions have been reported at this site.

## 3. Discussion

We describe an uncommon presentation of the recurrent nevus phenomenon developing within a keloid scar. Recurrent nevi most frequently arise following an excision of a primary nevus [2]. The recurrent nevus was once thought to be a malignant transformation of melanocytes occurring by an unknown mechanism, especially after electrodessication [2]. However, no studies to date provide strong evidence of malignant proliferation developing after the excision of a benign melanocytic lesion. The challenge in this case was the lack of a prior, clinically evident melanocytic lesion at the site. Importantly, the small nature of this melanocytic lesion may have contributed to the lack of visualization during the physical exam performed by the clinician. Moreover, the keloid lesion was recognized and excised by a general surgeon, who may not routinely conduct meticulous skin examinations such as that commonly performed in dermatology. 

Development of a recurrent nevus in the absence of any prior pigmented lesion is an unusual phenomenon and does not necessarily fit within the traditional definition. Various mechanisms leading to the development of the melanocytic proliferation above the keloid scar can be considered. First of all, we can postulate that a small melanocytic lesion, below the limit of clinical detection, may have been present prior to the piercing procedure. Therefore, the small melanocytic lesion, if pre-existing, may have been further developing at the top of the keloid scar after the piercing procedure and keloid development. Alternatively, the melanocytic lesion may have been induced due to a mass effect of the underlying proliferation of the keloid scar. In this scenario, the mechanical irritation of melanocytes and disruption of the dermal–epidermal junction would have exposed melanocytes to dermal proteins resulting in aberrant activation and proliferation [11]. Another hypothesis is that the growth factors produced within the keloid tissue may have contributed to the induction of this lesion. Finally, the piercing procedure may have induced a growth factor signaling cascade, similar to that seen after an excision procedure, inappropriately leading to the melanocytic proliferation. 

Melanocytes are hypothesized to play a role in pathological scar formation and have previously demonstrated an ability to upregulate fibroblast activity and increase collagen production primarily through an upregulation of transforming growth factor-beta (TGFβ) [11]. Therein, the presence of a recurrent nevus phenomenon in the absence of a primary nevus is plausible. An atypical melanocytic proliferation occurring within a keloid scar can be somewhat commensurate to the similar phenomenon of follicular basal cell hyperplasia developing over a pre-existing dermatofibroma, which is also characteristically benign and results from a dysregulation in intercellular communication [15,16]. 

Benign and malignant melanocytic lesions developing within a keloid scar are rare [13,14]. To add further complexity to the diagnosis, the recurrent nevus phenomenon must be differentiated from reactive melanotic pigmentation in scars [17]. Distinguishing a benign melanocytic lesion from a melanoma can be challenging especially when a benign melanocytic proliferation develops at a special site, such as the ear, and shares histological features with a melanoma. For example, in a report by Ardakani et al., development of clinically concerning pigmented lesions over areas recently affected by bullae in Stevens–Johnson syndrome prompted clinical concern for melanomas [18]. The atypical melanocytic proliferation exhibited histologic features resembling melanoma in situ because of the single-cell pagetoid spread, but this atypical melanocytic proliferation was overlying and was confined within the limits of a dermal scar and thus it was ultimately diagnosed as consistent with a recurrent nevus [18]. 

Similar occurrences of atypical, benign melanocytic lesions have been reported in other bullous disorders such as Hailey–Hailey disease [7]. Noor et al. describe a case of a pigmented lesions developing within a difficult-to-treat area of Hailey–Hailey disease [7]. Histological examination of the lesion revealed characteristics of the recurrent nevus phenomenon including, irregular junctional proliferation of melanocytes in nests as well as poorly nested melanocytes with pagetoid spread. Initially, the diagnosis of a recurrent nevus was overlooked in this case as there was a lack of clinical context describing Hailey–Hailey. Having learned that the patient had areas of recalcitrant Hailey–Hailey, a more appropriate diagnosis was given [7]. In the case of our patient, the knowledge of the previous piercing procedure and subsequent keloid formation allowed for an appropriate recognition and diagnosis of the recurrent nevus phenomenon arising above a keloid scar. This scenario further confirms the importance of knowing the clinical context in order to ultimately differentiate a benign recurrent nevus from a melanoma in situ. 

The shared histologic features present in the recurrent nevus phenomenon and malignant melanoma pose a relevant diagnostic challenge for dermatopathologists. Cellular features found on histology when evaluating the recurrent nevus phenomenon vary [5]. It is important to be aware of the overlapping and differentiating features of the recurrent nevus phenomenon and malignant melanoma. However, in cases where the diagnosis remains equivocal, additional tools such as PRAME and FISH (fluorescence in situ hybridization) may aid in an accurate diagnosis [19]. PRAME is a cancer-testis antigen that is highly expressed in melanocytes of melanoma but is absent or only expressed at low levels in benign melanocytes. Immunohistochemical staining for PRAME may be utilized in conjunction with melanocytic markers to aid in the differential diagnosis of ambiguous melanocytic lesions as a positive PRAME staining in a melanocytic lesion suggests a higher likelihood of malignancy in the right morphologic context [19]. 

Before confirmation of a diagnosis with a negative PRAME staining in this case, there were many histologic features that, when carefully evaluated, were pointing to a benign lesion. These included the minimal presence of pagetoid scatter (that was also somewhat confined to the very center of the lesion) and the lack of cytologic variability across the whole melanocytic proliferation. Importantly, the melanocytic lesion did not spread beyond the borders of the keloid scar. These features ultimately favored a benign diagnosis.

Currently, no broadly accepted guidelines or appropriate intervention algorithm exist for the management of a recurrent nevus. In this case, the lesion was completely narrowly excised and no recurrence has been recorded to date. We believe that in these cases, a conservative approach, with a recommendation for further resection in the event of recurrent pigmentation, should be considered the best management approach. Following complete excision of the lesion and confirmation of diagnosis, no recurrence has been reported. 

## 4. Conclusions

The recurrent nevus phenomenon may develop within a keloid scar in the absence of a previously existing pigmented lesion. Utilization of immunohistochemical techniques such as PRAME staining may be particularly beneficial in ambiguous cases. Appropriately recognizing this atypical melanocytic proliferation as a benign condition is important in reducing overtreatment.

## Data Availability

Not applicable.
